# Enhanced Clean-In-Place Monitoring Using Ultraviolet Induced Fluorescence and Neural Networks

**DOI:** 10.3390/s18113742

**Published:** 2018-11-02

**Authors:** Alessandro Simeone, Bin Deng, Nicholas Watson, Elliot Woolley

**Affiliations:** 1 Intelligent Manufacturing Key Laboratory of Ministry of Education, Shantou University, Shantou 515063, China; simeone@stu.edu.cn (A.S.); 17bdeng@stu.edu.cn (B.D.); 2 Department of Chemical and Environmental Engineering, University of Nottingham, Nottingham NG7 2RD, UK; Nicholas.Watson@nottingham.ac.uk; 3 Centre for Sustainable Manufacturing and Recycling Technologies (SMART), Loughborough University, Loughborough LE11 3TU, UK

**Keywords:** monitoring, resource efficiency, fluorosensing, image processing, neural network

## Abstract

Clean-in-place (CIP) processes are extensively used to clean industrial equipment without the need for disassembly. In food manufacturing, cleaning can account for up to 70% of water use and is also a heavy user of energy and chemicals. Due to a current lack of real-time in-process monitoring, the non-optimal control of the cleaning process parameters and durations result in excessive resource consumption and periods of non-productivity. In this paper, an optical monitoring system is designed and realized to assess the amount of fouling material remaining in process tanks, and to predict the required cleaning time. An experimental campaign of CIP tests was carried out utilizing white chocolate as fouling medium. During the experiments, an image acquisition system endowed with a digital camera and ultraviolet light source was employed to collect digital images from the process tank. Diverse image segmentation techniques were considered to develop an image processing procedure with the aim of assessing the area of surface fouling and the fouling volume throughout the cleaning process. An intelligent decision-making support system utilizing nonlinear autoregressive models with exogenous inputs (NARX) Neural Network was configured, trained and tested to predict the cleaning time based on the image processing results. Results are discussed in terms of prediction accuracy and a comparative study on computation time against different image resolutions is reported. The potential benefits of the system for resource and time efficiency in food manufacturing are highlighted.

## 1. Introduction

Increasing concerns over hygiene in the food and pharmaceuticals processing industry, coupled with high levels of risk aversion to cross contamination of foods (particularly allergens), emphasizes the importance of system cleaning within the food production industry. Production systems regularly contain a number of process tanks (e.g., for mixing, pasteurization, chilling) connected by many meters of pipework, heat exchangers and pumps. Historically, such systems were cleaned manually, requiring disassembly and scrubbing/rinsing, which was time consuming, introduced the potential for contamination and increased the likelihood of damage to the system.

Modern systems rely on a technique called clean-in-place (CIP), which allows the cleaning of the various sections of the production system without any disassembly [1]. Such systems may take on many forms, but often utilize spray balls in the larger volume components (i.e., tanks) and high flow rates of fluid in the small internal volume components (i.e., pipework and heat exchangers), in combination with various cleaning and sanitizing fluids (e.g., caustic soda) at elevated temperatures [2].

CIP systems are highly effective at removing system fouling and are suitable for automation: consequently, they are employed extensively in the majority of modern food and pharma production plants. Cleaning can account for as much as 70% of a food and beverage processer water use [3]. Such is the prevalence and resource intensity of the technique, it is imperative that the process be controlled as optimally as possible. Currently, there is a lack of real-time in-process monitoring that could enable effective control of the cleaning process parameters and cleaning durations and hence reduce resource consumption and increase the amount of time the production plant can spend producing.

The research reported in this paper is concerned with the design, implementation and data processing of an optical monitoring system for tanks and other large volume components. Following a review of current monitoring capabilities and fouling assessment techniques, a system of hardware for detection of residual foodstuffs under non-ideal conditions is described. Laboratory scale results are obtained from a purpose-built CIP test rig and an image processing procedure described. The results are analyzed using neural networks to indicate how cleaning times could be reduced within an industrial application. The paper concludes with a discussion of future developments of the technology and it’s applicably to real industrial environments. 

## 2. Literature Review

Certain molecular structures will, under excitation by appropriate high energy (short wavelength) light, experience electronic excitation. Such excitation can lead to photoluminescence either as fluorescence (‘immediate’ emission of photons) or phosphorescence (delayed emission of photons). Strictly the differentiation between fluorescence and phosphorescence depends on whether the excited electrons experience a change in spin [4]. In this paper, the term fluorescence to mean is used to mean immediate emission of photons regardless of the excited electronic state.

**Figure 1:**
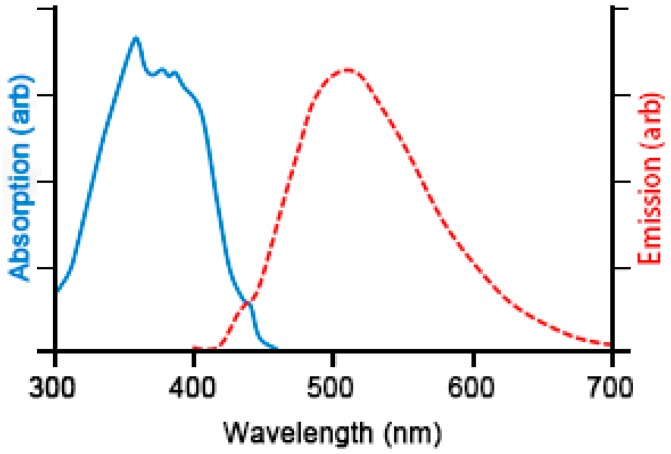
Illustrative chart of excitation and emission spectra of 3-hydroxy-DL-kynurenine (3-OHK). Adapted from [5].

Fluorosensing, the sensing of fluorescence, is a useful technique to identify the presence of certain chemicals within a sample, as it allows excitation by a narrow or broad range of wavelengths of light and detection of light emission in another part of the spectrum. Typically, but not exclusively, excitation occurs in the ultraviolet range of the spectrum whilst detection occurs within the visible wavelength range (see Figure 1). This allows for a decoupling of excitation and detection systems to reduce false positive signals. 

Fortuitously, many natural and synthetic chemical structures fluoresce under excitation by an appropriate wavelength of UV light. Indeed it has been reported that dairy products contain many important fluorophores which are utilizable for fluorescence spectroscopy [6]. These fluorophores include the aromatic aminoacids—tryptophan, tyrosine and phenylalanine in proteins [7,8], vitamin A and B2, reduced nicotinamide adenine dinucleotide derivatives of pyridoxal and chlorophyll, some nucleotides and various other compounds that may be found at a low levels of concentration in food. Thus this phenomenon has found widespread application across many areas of research and industry: agriculture, forensics, fraud prevention, process monitoring, entertainment, amongst many others [9,10,11].

In the field of plant physiology excitation wavelength in the UV-A region (315–400 nm) are typically used to excite certain species under investigation [12]. Fluorescence emissions are classified as being in either the blue-green (400–630 nm) or red-far red (630–800 nm) regions and the intensity of individual peaks can be attributed to the concentration of specific chemical components, indicating the health of those plants. It is possible also to consider the ratio of intensities between individual peaks to monitor the health of plants.

For applications where fluorescence intensity is low, where there is excessive external light, or where partial reabsorption occurs (due to overlap of absorption and emission spectra) laser sources can be used for excitation either as a focused or unfocused beam [13]. 

Within manufacturing, fluorosensing has been used for the detection of grease (conjugated double bond hydrocarbons (alkenes)) on mechanical components during cleaning processes [14]. Issues arise when trying to monitor the amount of fouling remaining since there is a saturation of signal above certain thicknesses, making it difficult to determine the volume of fouling remaining.

### 2.1. Image Processing

In order to enhance imaging (spatial assessment of specimens) a range of filters have been implemented to better differentiate between the different emission spectra from various chemical structures present [15], although this has the requirement of a mechanical filter change which extends sensing time. A more simple method of image analysis has been described which utilises the red, green and blue (RGB) components of a color image captured by either a Complimentary Metal Oxide Semiconductor (CMOS) or Charged Coupled Device (CCD) sensor [16,17].

Nedbal et al. [18] developed an image processing technique to detect variations in Chl fluorescence parameters over the surface of a lemon fruit for predicting areas that will eventually exhibit visible damage. Wan et al. applied deconvolution technique to fluorescence images to retrieve the high-precision plant fluorescence lifetime of single pixel point in plant continuous fluorescence images [19]. Segmentation based image processing techniques were utilized by Shrivastava et al. for the detection of Staphylococcus aureus in a culture-free, rapid, quantitative manner from minimally processed liquid samples using aptamer-functionalized fluorescent magnetic nanoparticles [20].

Image processing techniques have been widely utilized to generate input features for prediction tasks via neural networks.

Lin et al. [21] applied artificial neural network (ANN) to multi-spectral data analysis and modelling of airborne laser fluorosensor in order to differentiate between classes of oil on water surface. Peleato et al. [22] investigated the use of fluorescence data coupled with neural networks for improved predictability of drinking water disinfection by-products (DBPs).

Cancilla et al. carried out a fluorescence study using various light sources at 400 nm and ANNs to assess the concentration of ionic liquid aqueous solutions in a wide range of concentrations [23].

Huang et al. proposed a concentration–synchronous–matrix–fluorescence (CSMF) spectroscopy combined with 2D wavelet packet and probabilistic neural network (PNN) for source recognition of crude oil and petroleum products samples [24]. 

With reference to plant disease detection, a comprehensive, Golhani et al. [25] reviewed advanced Neural Network (NN) techniques available to process imaging and non-imaging hyperspectral data.

### 2.2. Applications in Food Processing

Fluorosensing has been investigated for use in food safety applications notably for the detection of fecal residues on fresh produce [26,27] and for tumors on chicken carcasses [28]. A review of the technology is provided in [29]. There has also been some previous activity within the quality assurance in the dairy industry, with the authors of [30] showing that front-face fluorescence (FFFS) can be used to track the Mailliard reaction during the processing of milk. Similarly fluorosensing has been used to monitor the effect of both packaging and exposure to light on the oxidation of yogurt [31] monitoring the presence of both tryptophan and riboflavin. A review of FFFS for monitoring dairy based food products in provided in [6]. 

Alternatively, UV light detection methods, are particularly used for the detection of residual cells and soiling on industrial surfaces [32].

UV induced fluorescence has been used for the detection of residual cells and soiling on industrial surfaces [33] with little change in findings when microorganisms are present. The molecular configuration of organic material allows some organic residues to fluoresce when illuminated by UV light [33]). Thus, UV light may be used to detect residual soil when work surfaces are illuminated by an appropriate wavelength; highlighting areas in an industrial plant that need be cleaned more intensively.

Fluorosensing is evidently a highly versatile technique for detecting the presence of certain chemical components, and can even be used to determine different states of the same fluorophores. The application of fluorosensing within CIP systems provides a number of challenges, which are detailed in the remainder of this manuscript.

## 3. Materials and Methods

In this section, an experimental two tank CIP system is described endowed with a UV illumination source and CMOS camera alongside sample preparation and image processing procedures. The system is designed to simulate an industrial food processing and CIP system. In this investigation, images were captured in real-time and were later post-processed.

### 3.1. Two Tank System

**Figure 2:**
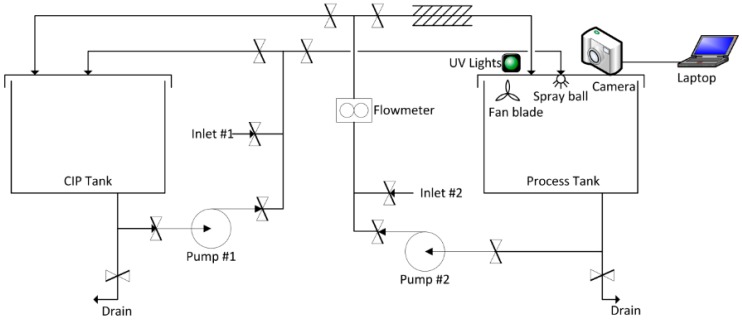
Experimental rig scheme.

An industrial grade two tank stainless steel system was constructed, incorporating one process tank and one CIP tank and interconnected by two centrifugal pump driven circuits as shown in Figure 2. Each tank has a 600 mm internal diameter, with 315 mm height and a 50 mm insulated wall and dished base with a centrally located anti-vortex drainage hole. The process tank was fitted with an 18 mm Tanko S30 dynamic spray ball located centrally in the lid. Piping for the system was SWG 25.4 × 1.6 mm fitted with manually operated butterfly valves.

**Figure 3:**
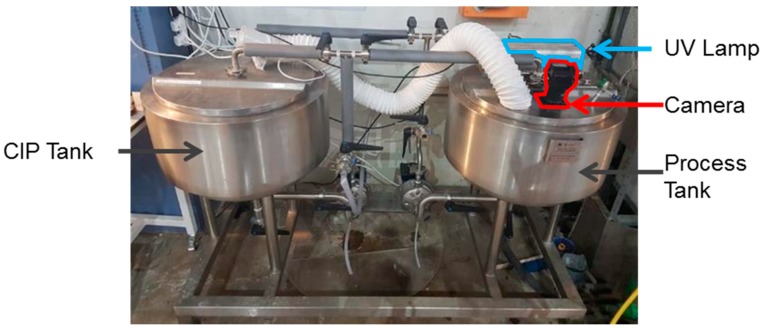
Experimental rig.

**Figure 4:**
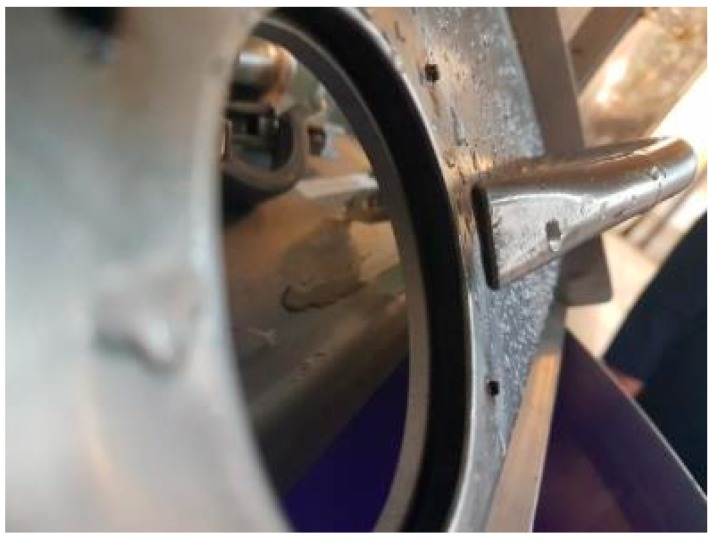
Air knife.

In order to improve visibility in the process tank (for the optical detection system) an ‘air knife’ was fabricated to blow compressed air laminar to the exposed camera lens (see Figure 2, Figure 3 and Figure 4), and an extractor fan (50 L s^−1^) installed to remove some proportion of steam and vapor from the tank whilst the spray ball was in operation.

### 3.2. Optical System

**Figure 5:**
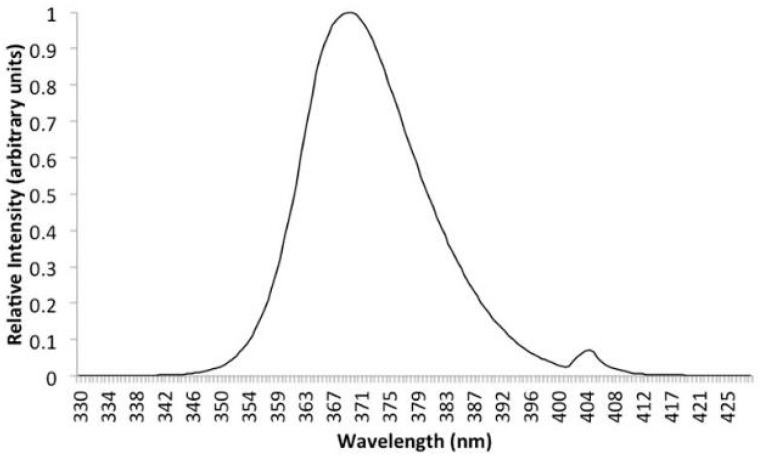
Intensity vs. wavelength.

The process tank was optically isolated from the surrounding laboratory to reduce unwanted signal during monitoring. Sample excitation was provided by a dual 18 W 370 nm (nominal) fluorescent lamp installed toward the rear of the lid. A spectral emission for the lamp is shown in Figure 5.

Images were acquired using a Nikon D330 DSLR and a 10–20 mm F4-5.6 EX DC HSM wide angle zoom to maximize the visual field. The camera was mounted using an adaptor which kept the front of the lens flush with the upper surface of the process tank lid. The zoom was manually adjusted to optimize image clarity and then fixed for the duration of the experimental investigation. Other photographic parameters, established experimentally to provide a high-quality image, were also kept constant:

- ISO sensitivity: 12,800
- F-Stop: F/4
- Exposure time: 1/100 s

A remote shutter control was used to prevent misalignment of the camera during image capture.

### 3.3. Fouling Preparation

For this experimental campaign, white chocolate was utilized as fouling medium as using the described two tank system. This particular medium was selected as it had a sufficiently long cleaning time within the experimental rig such that a series of high-quality images could be captured for fouling level analysis. The white chocolate is also representative of many types of food fouling that occur in food processing industries having both high fat and sugar content. For the brand of chocolate used, the nutritional composition is reported in Table 1.

**Figure 6:**
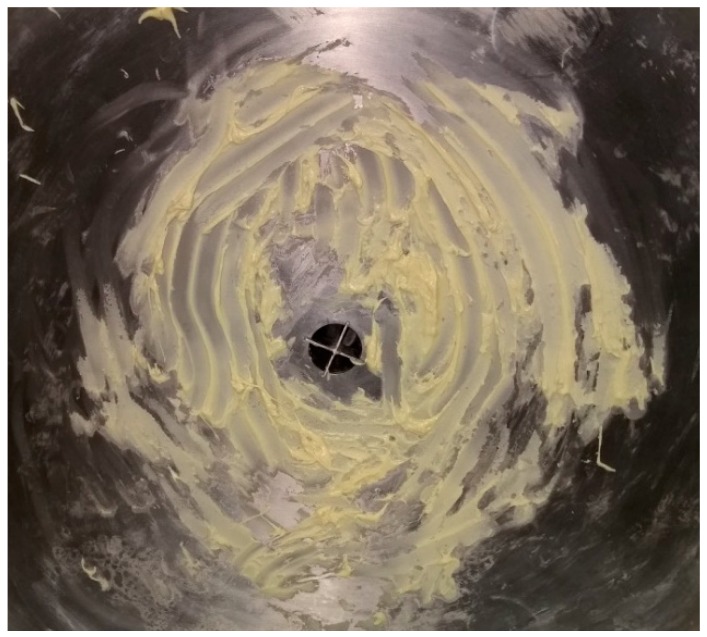
Deposited white chocolate fouling.

Before each fouling application, the process tank was manually scrubbed using a detergent and then thoroughly rinsed to ensure no fouling residues remained. The fouling was prepared by first gently heating 0.15 kg of white chocolate to melting point in a small receptacle before being manually spread by hand over the full inner surface of the dished base of the process tank (see Figure 6). Partial solidification of the fouling occurred due to contact with the cool tank wall.

### 3.4. Washing Cycles

The type of wash utilized in this investigation to remove the fouling from the process tank consisted in a hot wash using mains heated water (nominally 55 °C) held in the CIP tank and then pumped through the spray ball into the process tank and drained to the main sewer (H).

The cleaning cycle was operated until the tank was visibly clean. If the CIP tank emptied before the process tank was clean, the procedure was paused, and the CIP tank refilled before resuming the cleaning cycle.

Three experimental tests of fouling wash were carried out by repeating three times the above-mentioned procedure, generating three experimental datasets, D1, D2 and D3 respectively.

### 3.5. Image Acquisition

A baseline image for a clean tank illuminated by the UV lamp was recorded before each fouling application. Digital images where acquired during the cleaning cycles at 5 s intervals using a remote trigger. The image resolution adopted for this experimental campaign was 2000 × 2992 pixels corresponding to 6 MP images.

### 3.6. Image Processing

**Figure 7:**
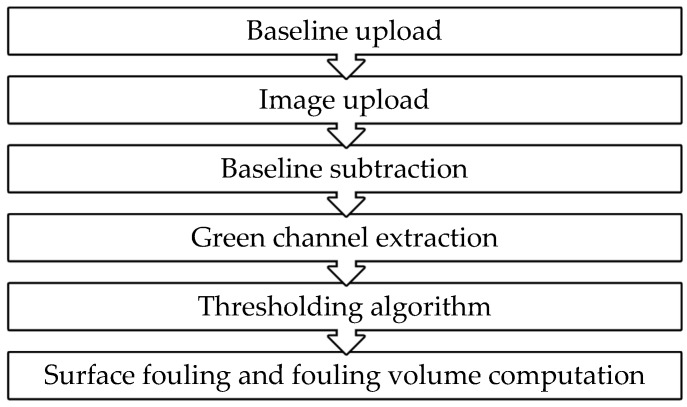
Image processing procedure.

The image processing procedure outline is reported in Figure 7.

**Figure 8:**
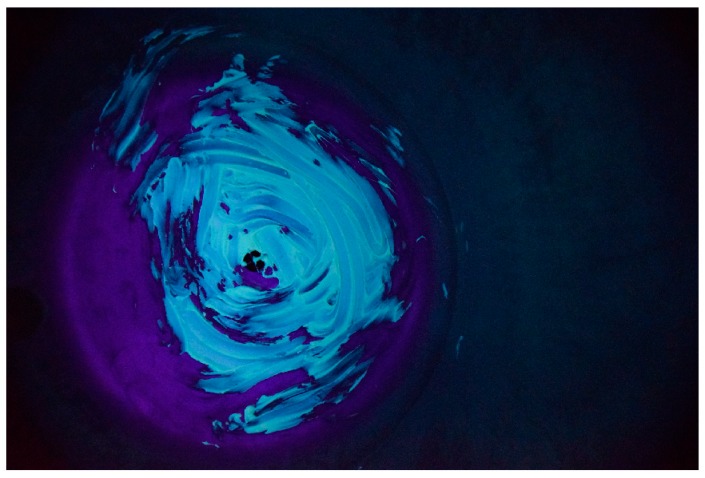
Raw image.

The software utilized for the image processing was Matlab^®^. The procedure starts by uploading the digital image to the software. The raw image appears as a 2000 × 2992 × 3 elements matrix, where the first two dimensions (2000 × 2992) represent the image resolution, and the third dimension (3) is represented by the three colors channels; red, green and blue (RGB) respectively. An example of the raw image is reported in Figure 8.

#### 3.6.1. Baseline Upload and Subtraction

**Figure 9:**
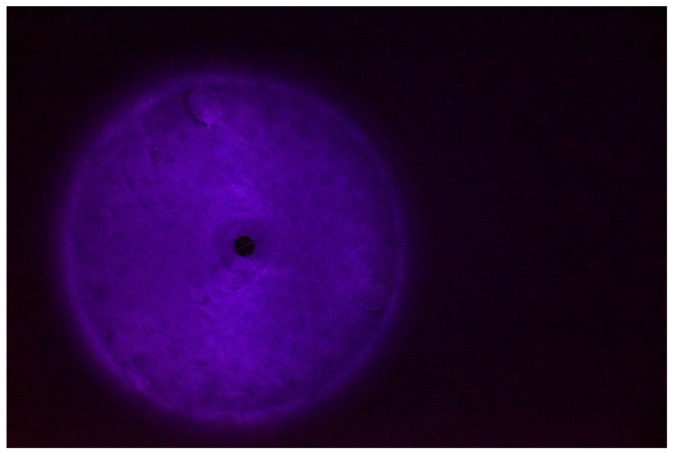
Baseline image.

An image of the clean tank was acquired prior to the fouling application and used as a baseline (example shown in Figure 9).

**Figure 10:**
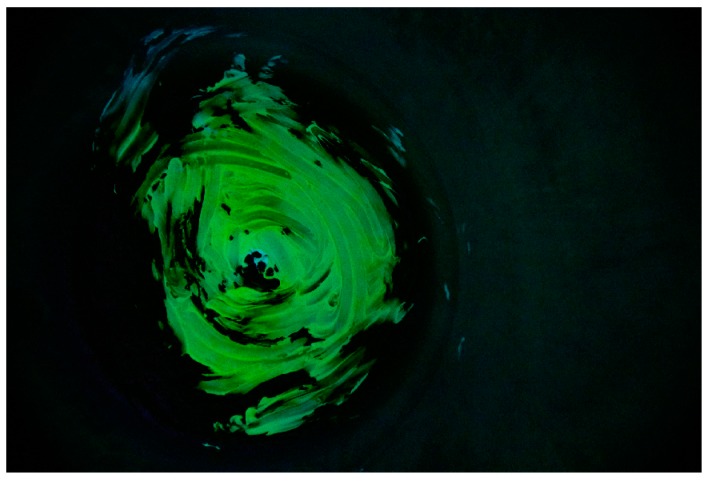
Subtracted image.

In order to remove background signal from unfouled areas of the tank an image subtraction [34,35] operation was then carried out, by subtracting the baseline image from the raw image. The resulting image can be visualized in Figure 10.

**Figure 11:**
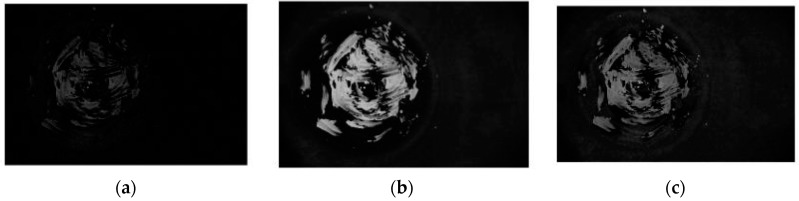
Digital image channels: (**a**) red channel; (**b**) green channel; (**c**) blue channel.

The background subtracted image still contains the three component RGB channels. Performing a channel separation operation to the image reported in Figure 11 it is apparent that most of the information on the fouling is contained within the green channel as is expected [12]. There is a danger that the blue channel could contain false positive signal from the emission of the UV lamp as the response of the camera overlaps with the emission spectra (as can be seen in Figure 11), whilst the red channel provides little signal. Thus, in order to simplify processing and reduce the potential for false positive signal the remaining image processing steps were performed using only the green channel image, essentially filtering out the blue and red wavelength ranges.

Once the green channel has been isolated, thresholding must be carried out to determine which signal can be classed as fouling and which can be regarded as a ‘clean’ area of the tank. A range of thresholding techniques are discussed and their suitability evaluated.

#### 3.6.2. Otsu Method

The Otsu thresholding algorithm [36,37] aims at dividing pixels of an image into two segments $S_{0}$ and $S_{1}$ (e.g., objects and background) at intensity level T. Let $\sigma_{W}^{2}$, $\sigma_{B}^{2}$ and $\sigma_{T}^{2}$ be the within-class variance, between-class variance, and the total variance, respectively. Optimal threshold is obtained by minimizing $\sigma_{W}^{2}$. Following is the relation between different class variances.

(1) $$\alpha =\frac{\sigma_{B}^{2}\;}{\sigma_{W}^{2}}$$

Thus, the optimal threshold *T* is obtained by maximizing α and can be defined as

(2) $$\;T=\underset{t}{\max\alpha }$$

where:

(3) $$\sigma_{W}^{2}=\omega_{0}\sigma_{0}^{2}+\omega_{1}\sigma_{1}^{2}$$

(4) $$\sigma_{B}^{2}=\omega_{0}{\left(\mu_{0}-\mu_{T}\right)}^{2}+\omega_{1}{\left(\mu_{1}-\mu_{T}\right)}^{2}$$

(5) $$\sigma_{T}^{2}=\sum_{i=1}^{L}{\left(1-\mu_{T}\right)}^{2}P_{i}$$

(6) $$\omega_{0}=\sum_{i=0}^{t}P_{i},\;\;\omega_{1}=1-\omega_{0},\;\;\mu_{1}=\frac{\mu_{T}-\mu_{t}}{1-\mu_{0}},\;\;\mu_{0}=\frac{\mu_{t}}{\omega_{0}}$$

(7) $$\mu_{t}=\sum_{i=0}^{t}iP_{i},\;\mu_{T}=\sum_{i=0}^{L-1}iP_{i},\;G=\left\{0,1,2,\ldots ,L-1\right\}$$

where $n_{i}$ is the total number of pixels with grey-level, i and *n* is the total number of pixels in the given image defined as $n=\sum_{i=0}^{L-1}n_{i}$. Probability of grey-level $P_{i}$ is defined as $P_{i}=\frac{n_{i}}{n}$.

#### 3.6.3. Iteration Method

The iteration method [38] is made of a series of iterative steps and it is initialized by selecting an initial threshold value computed using the above-mentioned Otsu’s method. The iterative steps are reported below:

(a) Choose an initial estimate threshold value T obtained by Otsu’s method [36].
(b) Compute the minimum and maximum grey values of the digital image, respectively min and max. Use $T_{1}=0.5\;\times \left(\min+T\right)$ to perform an image segmentation into two sets of pixels, specifically $G_{1}$ (including pixels whose values are lower than $T_{1}$ and $G_{2}$ (made of pixels higher than $T_{1}$ but lower than T).
(c) Calculate the average brightness $g_{1}$ of $G_{1}$, and the average brightness $g_{1}$ of $G_{2}$
(d) Calculate $T_{1}$ where $T_{1}=0.5\;\times \left(g_{1}+g_{2}\right)$.
(e) Repeat step (b) to step (d) until difference between the current $T_{1}$ and the previous one is less than 0.5.
(f) Use $T_{2}=0.5\;\times \left(T_{1}+\max\right)$ to perform an image segmentation into two sets of pixels, specifically $G_{3}$ (including pixels whose values are lower than $T_{2}$ but higher than $T_{1}$ and $G_{4}$ (made of pixels higher than $T_{2}$ but lower than max).
(g) Calculate the average brightness $g_{3}$ of $G_{3}$, and the average brightness $g_{4}$ of $G_{4}$
(h) Calculate $T_{2}$, where $T_{2}=0.5\;\times \left(g_{3}+g_{4}\right)$.
(i) Repeat step (f) to step (h) until difference between the current $T_{2}$ and the previous one is less than 0.5.
(j) $T_{1}$ and $T_{2}$ are the final threshold values to be used for the image segmentation.

#### 3.6.4. Maximum Entropy 1D

The Maximum Entropy 1D approach [39] is utilized to select a threshold such that the information available in the two grey-level distributions of the foreground and the background is maximized. The information is measured by the entropy. The appropriate steps are explained below:

Let $f_{1},f_{2},\ldots ,f_{m}$ represent the observed grey-level frequencies (histogram). The expression for the probabilities (percentage of occurrence of a specific grey level), $p_{i}$, becomes:

(8) $$p_{ij}=\frac{f_{ij}}{N^{2}},\;\sum_{i=1}^{m}f_{i}=N^{2},\;i=1,2,\ldots m\;$$

where $N^{2}$ is the total number of pixels in the image and *m* is the number of grey levels in the histogram. It is reasonable to assume that only foreground values make up clusters and the background values consist of noise. Setting the grey levels above a threshold value (s) equal to 1 and the rest equal to 0, results in a binary image. By maximizing the entropy criterion, with respect to *s*, the threshold is obtained. The entropy criterion, ψ(s), is defined by:

(9) $$T=\underset{s}{\max}\;\left(lnP_{s}\left(1-P_{s}\right)+\frac{H_{s}}{P_{s}}+\frac{\left(H_{m}-H_{s}\right)}{\left(1-P_{s}\right)}\right),$$

where

(10) $$\;H_{s}=-\sum_{i=1}^{s}\left(p_{i}\ln p_{i}\right)$$

(11) $$P_{s}=\sum_{i=1}^{s}p_{i}$$

(12) $$H_{m}=-\sum_{i=1}^{m}\left(p_{i}\ln p_{i}\right)$$

(13) $$P_{m}=\sum_{i=1}^{m}p_{i}$$

#### 3.6.5. Maximum Entropy 2D

The first step in the Maximum Entropy 2D procedure [40] is to divide the grey level and its average into *m* values. The algorithm computes the average grey-level value of the neighborhood for each pixel. In this way a pair is obtained, made of the pixel grey level and the neighborhood average. Each pair is assigned to a 2-dimensional bin. The total number of bins results to be m×m and the total number of pixels to be tested is N×N. Subsequently, the algorithm calculates the joint probability mass function, $p_{ij}$ as the ratio of the frequency $f_{ij}$, of a pair (i, j) and the total number of pixels, $N^{2}$:

(14) $$\;p_{ij}=\frac{f_{ij}}{N^{2}}\;\;i,j=1,\ldots ,m\;$$

Considering the foreground and background groups, respectively *A* and *B* with two different probability mass functions (PMF), if the threshold is located at the pair (s, t), then the total area under $p_{ij}\left(i=1,\ldots ,\;s\;\mathrm{and}\;j=1,\ldots ,t\right)$ is equal to one, being $p_{ij}$ in this region the conditional PMF. After a normalization process it is possible to compute the modified entropy for group *A*, *H*(*A*), defined as:

(15) $$\;H\left(A\right)=-\sum_{i=1}^{s}\sum_{j=1}^{t}\left(\frac{p_{ij}}{P_{st}}\right)\ln\left(\frac{p_{ij}}{P_{st}}\right)\;$$

where

(16) $$\;P_{st}=-\sum_{i=1}^{s}\sum_{j=1}^{t}p_{ij}$$

(17) $$\;H\left(B\right)=-\sum_{i=1}^{s}\sum_{j=1}^{t}\left[\frac{p_{ij}}{\left(1-P_{st}\right)}\right]\ln\left[\frac{p_{ij}}{\left(1-P_{st}\right)}\right]\;$$

(18) $$\;T=\underset{s,t}{\max}H\left(A\right)+H\left(B\right)\;$$

**Figure 12:**
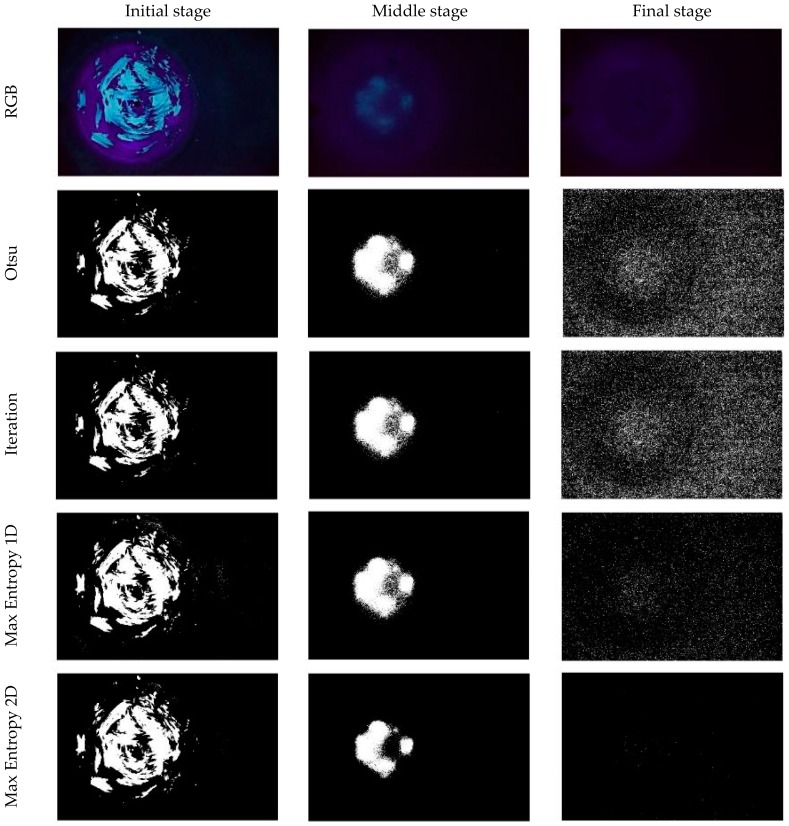
Thresholding methods comparison.

Figure 12 shows a comparison of the four thresholding methodologies for three sample images, acquired at the initial, middle and final stage of the washing cycle respectively.

Taking into account the Signal to Noise ratio (S/N) [34] computed throughout the washing cycle, and considering the average thresholding computation time, the Maximum Entropy 2D thresholding method was adopted in this work to estimate the surface fouling and fouling volume as detailed in the following sections.

### 3.7. Surface Fouling Computation

Once the image processing procedure is completed, it is possible to compute the surface and the volume of fouling in each image.

**Figure 13:**
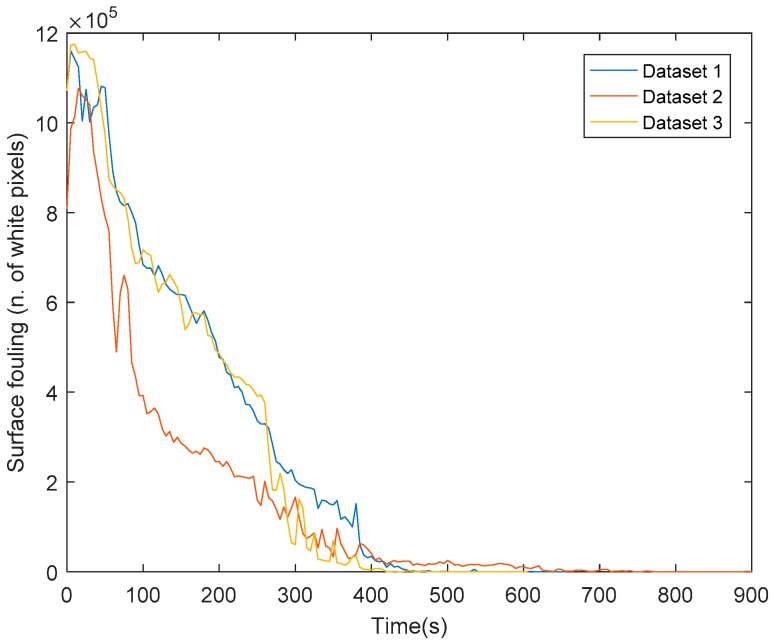
Surface fouling chart.

The surface fouling in each image was computed by summing all the white pixels resulted from the thresholding operation, precisely:

(19) $$SF=\sum_{i=1}^{n\cdot m}{\mathrm{pixel}}_{i},\;\mathrm{pixel}=1$$

where *n* and *m* are the digital image dimensions, and 1 represents the normalized value for white pixels. The surface fouling chart vs. time is reported in Figure 13 for all the datasets.

From the chart, it is possible to observe fluctuations in the decreasing trend of surface fouling, due to the water spray force, which spreads the chocolate lumps on a wider surface before they can be drained out.

### 3.8. Thickness and Volume Estimation

Previous works by the authors demonstrated that the fouling thickness is proportional to the pixel intensity within the digital image [16].

A fouling volume indicator can be calculated as the sum of white pixels multiplied by the pixel intensity of the corresponding pixel in the green channel.

(20) $$V=\sum_{i=1}^{n\cdot m}{\mathrm{pixel}}_{i}\times {\mathrm{Green}\;\mathrm{Intensity}}_{i},\;\mathrm{pixel}=1$$

**Figure 14:**
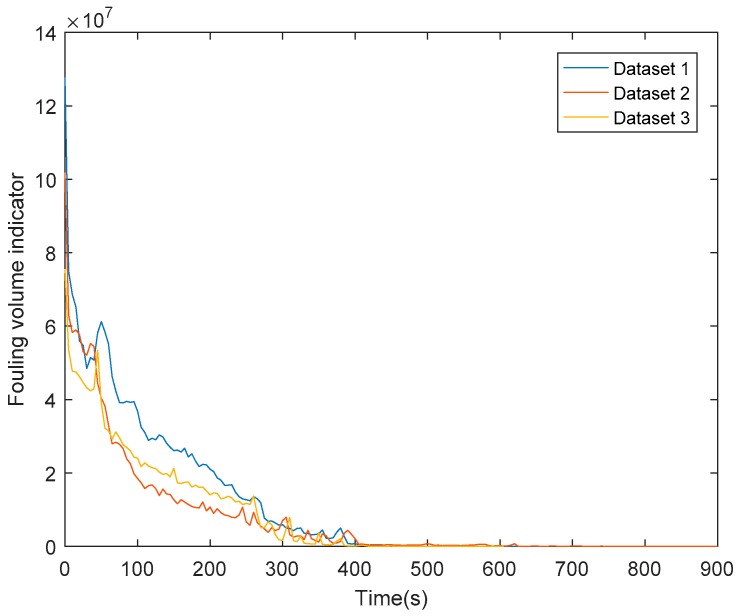
Fouling volume indicator chart.

The fouling volume indicator chart vs. time is reported in Figure 14 for all the datasets.

**Figure 15:**
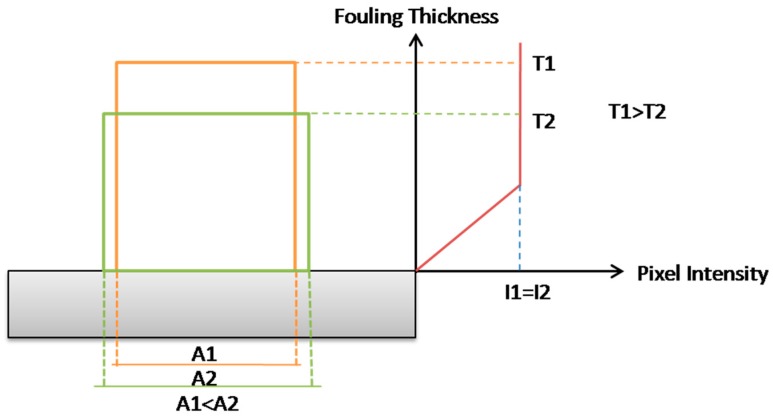
Pixel intensity vs. fouling thickness diagram.

The fluctuations in the fouling volume indicator chart are due to the proportionality of the fouling thickness to the pixel intensity, which is increasing until a certain value and then asymptotically floating [14,16] as schematically reported in Figure 15.

## 4. Intelligent Decision Making on Cleaning Time Prediction

In this section, a neural network-based decision-making support system was built with the aim of predicting the required cleaning time based on the fouling volume indicator estimation results obtained using the image processing and thresholding technique described in the previous sections. 

The fouling volume indicator over time was used as input to train different configurations of time series prediction neural networks, subsequently, the trained system was tested to each dataset to assess the cleaning time accuracy in terms of mean squared error and output element response.

A discussion on neural network architecture performance is reported to determine the best configuration in terms of training dataset and hidden layer nodes.

### 4.1. NARX Network

Nonlinear autoregressive models with exogenous inputs (NARX) are recurrent neural architectures [41,42], in which the feedback architectures come only from the output neuron instead of from hidden neurons.

NARX is an important class of discrete-time nonlinear systems that can be mathematically represented as:

(21) y(n+1) = f[y(n),…,y(n−dy+1);x(n−k), x(n−k−1),…,x(n−k−du+1)]

where x(n),y(n)∈ℜ denote, respectively, the input and output of the model at discrete time step *n*, while *du* ≥ 1, *dy* ≥ 1 and *du* ≤ *dy*, are the input-memory and output-memory orders, respectively. The parameter *k* (*k* ≥ 0) is a delay term, known as the process dead-time [43].

The nonlinear mapping f(·) is general unknown and can be approximated, for example, by a standard multilayer perceptron (MLP) network. The resulting connectionist architecture is then called a NARX network, a powerful class of dynamical models which has been shown to be computationally equivalent to Turing machines [44].

### 4.2. Architecture

Different training datasets were used, consisting of single datasets (D1, D2, D3), double datasets (D1 + D2, D1 + D3 and D2 + D3) and a triple dataset (D1 + D2 + D3). Five different hidden layer nodes configurations were used, consisting of 3, 6, 10, 15 and 20 hidden layer nodes respectively. The delay term was set to 2 samples for all the configurations.

The training algorithm adopted was the Bayesian Regularization [45], which minimizes a linear combination of squared errors and weights. It also modifies the linear combination so that at the end of training the resulting network has good generalization qualities.

The predicted output consists in the cleaning time, i.e., the time in which the fouling volume = 0.

**Figure 16:**
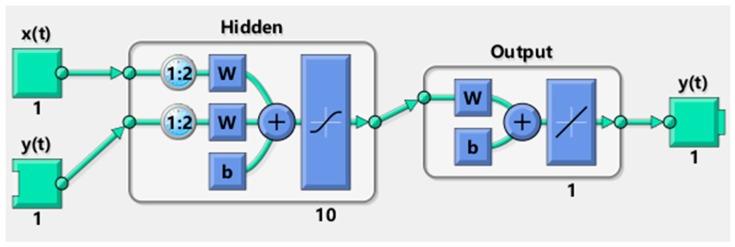
NARX Neural Network for single dataset training and 10 hidden layer nodes.

Figure 16 reports a NARX neural network scheme for a single dataset training and 10 hidden layer nodes as an example.

## 5. Results

Table 2 shows the results of the trained NARX networks tested on D1, D2 and D3. As a measure of NN performance, the Mean-Squared Error (MSE) [46] was considered. The results in bold represent the best configurations in terms of hidden layer nodes for each testing dataset.

In all the tests, the correlation coefficient was equal to 1, demonstrating a perfect fitting suitability.

**Figure 17:**
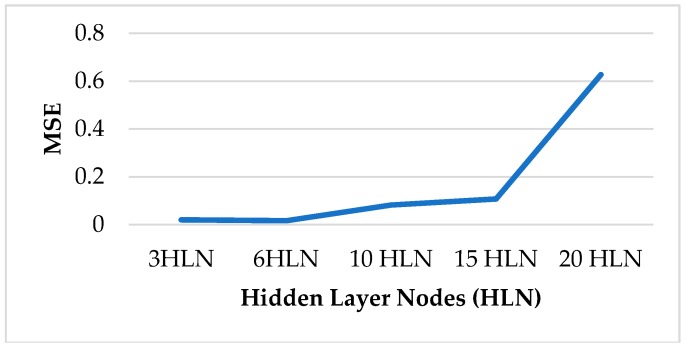
NARX Neural Network performance vs. hidden layer nodes.

Figure 17 shows a better performance (lower MSE) for smaller hidden layer nodes numbers, i.e., 3 and 6. Such result suggests that for this application a high number of hidden layer nodes leads to overfitting phenomena [47].

**Figure 18:**
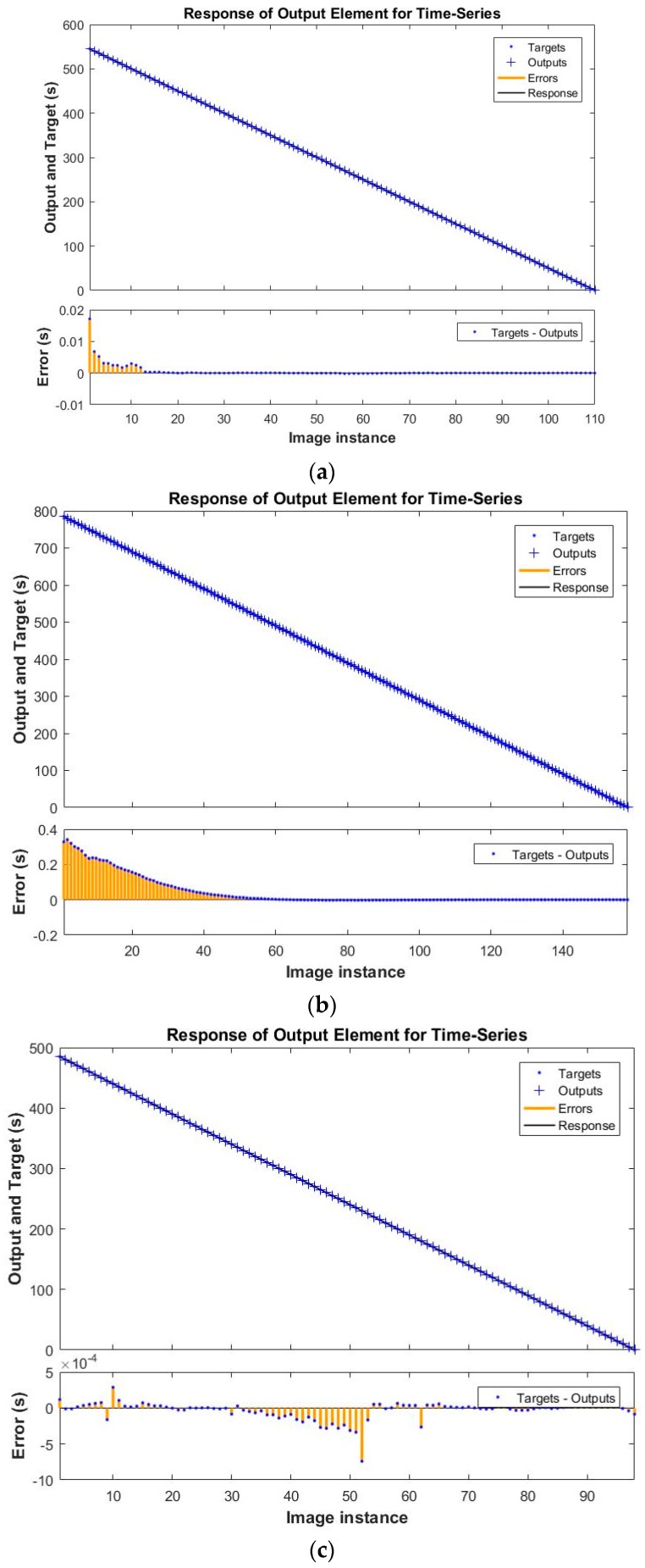
NARX Neural Network response of output element. Results refer to three best configurations in predicting cleaning time for Dataset D1 (**a**); D2 (**b**) and D3 (**c**).

Figure 18a–c shows the NARX Neural Network response (Cleaning Time) comparing the target (actual) to the output (predicted) for various configurations. In the initial phase, due to the limited number of samples, the prediction errors are slightly higher. Then, as the number of time series samples increases, the NARX response becomes more accurate and the prediction error decreases significantly. The extremely low error ranges (10^−1^ to 10^−4^ s) show a great suitability for industrial applications.

### Computation Time vs. Resolution

An important aspect in the design and implementation of a monitoring system is represented the computation and response time, which needs to be considerably lower than the data sampling time. 

In this study, an acquisition rate of one image every five seconds was adopted with 6 MB resolution images. For an industrial application this rate may be not adequate, and, on the other hand, such high resolution can result redundant and therefore costly. 

For these reasons, a comparative study is proposed here to evaluate the computation time for various image resolutions, and the related image processing accuracy is assessed in terms of determination coefficient as compared to the original resolution.

Four different image resolutions were used for this comparative study.

- L = Original resolution = 2000 × 2992 px
- M = 1500 × 2244 px
- S = 1000 × 1496 px
- XS = 500 × 748 px

**Figure 19:**
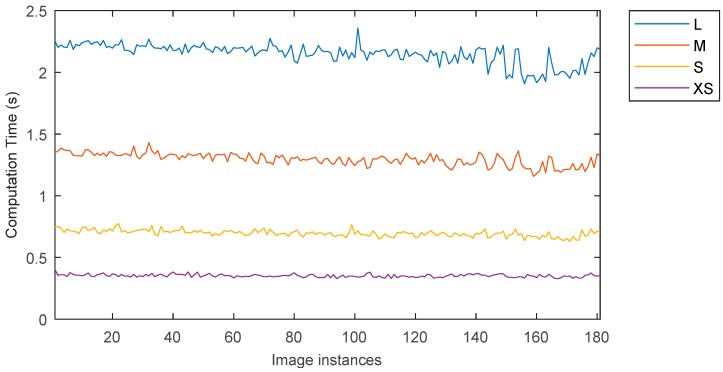
Computation time vs. image resolutions for D2 dataset.

The elapsed time to carry out the full image processing methodology reported in Figure 19 was recorded for all the image resolutions considered. The average computation time per image resolution is reported below.

- L = 2.1498 s
- M = 1.2953 s
- S = 0.6962 s
- XS = 0.3518 s

Considering the sampling frequency, the proposed image processing methodology is suitable for the clean-in-place monitoring system for all the resolutions range.

In order to evaluate the system accuracy for the different image resolutions, a comparison was carried out using the linear regression coefficient computed between the surface fouling results obtained with the target dataset, i.e., large resolution (L: 2000 × 2992 px) and the output datasets, i.e., the ones obtained with the other resolutions.

The coefficient of determination of a linear regression model is the quotient of the variances of the target values and output values of the dependent variable [48]. 

Denoting $y_{i}$ as the observed values of the dependent variable, $\hat{y}$ as its mean, and ${\hat{y}}_{i}$ as the target value, then the coefficient of determination is:

(22) $$\;R=\sqrt{\frac{\sum {\left({\hat{y}}_{i}-\hat{y}\right)}^{2}}{\sum {\left(y_{i}-\hat{y}\right)}^{2}}}\;$$

**Figure 20:**
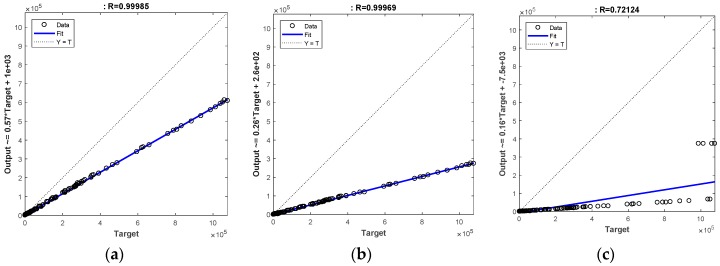
Coefficient of determination between original resolution (L) and M (**a**); S (**b**) and XS (**c**) resolutions.

The results are plotted in Figure 20.

For M and S resolutions, results show a very strong linear correlation with the original resolution, which means that the fouling detection can be carried out using a lower resolution without any significant loss in detection performances. A lower linear correlation coefficient was found for the XS resolution, indicating a worse performance.

## 6. Concluding Discussion

Fluorosensing is a well-recognized technique for assessing the condition of biological matter. In this research it has been investigated with regard its applicability to monitor cleaning processes within the food manufacturing industry

The use of purpose-built stainless steel two tank system enables the simulation of industrial cleaning processes that provided suitable data for further analysis. The experimental procedure described, relying on UV induced fluorescence, allows for the capture of images representative of those which might be obtained from an industrial application which can then be post processed.

An image processing procedure was developed which essentially isolated the green channel from the RGB images, as this channel was considered to contain the highest levels of true positive signal for the white chocolate samples investigated. It may be the case that other types of fouling sample may have different emission spectra and so, utilizing an RGB camera, the different (or a combination) of channels might be more suitable for analysis.

As part of the image processing procedure, a range of thresholding methodologies were investigated with Max Entropy 2D found to be the most successful (best S/N) of those investigated. The processing procedure as established was then applied to a number of sets of images throughout the entire cleaning cycles executed on the test rig. In this way, it was possible to consider disturbance factors, such as the false positive occurring due to the fouling fluorescence reflection on the steel bottom and side surfaces, throughout the entire washing cycle. In addition to this, the randomization of the initial manual fouling application, along with three experiments repetitions guarantee the robustness and repeatability of the described process analysis, as confirmed by the small variability in terms of surface fouling and fouling volume trends as shown in Figure 13 and Figure 14. 

An image processing procedure has therefore been developed that successfully enables the analysis of a state of fouling of food production vessel. This in itself would be useful within industrial applications allowing for an additional mechanism to assess and validate the cleanliness of production equipment. However, the sensor architecture described has demonstrated the ability capture and process images in near-real time, which would enable the continuous monitoring of cleaning processes. The technique therefore allows an assessment of fouling during cleaning and can alert on operator to a situation where a sufficient level of cleaning has been achieved or where cleaning is insufficient in a given time. The first of these options has the potential to shorten cleaning times (and associated resource consumption) while the latter has the potential to improve food safety.

A further capability is described in this research. The implementation of neural networks has been demonstrated to allow the prediction of the point in time when a ‘clean’ state will be achieved. The capability is important as it allows manufacturers to further capitalize on the benefits of the system by preparing the follow-on production batch to be ready for the point at which the system reaches its clean state.

The research presented has been demonstrated at the laboratory scale. There is a need to develop the optical hardware into a system that is satisfactory for industrial scale applications (e.g., size and robustness) and to develop a control system that is compatible with existing CIP installations. However, this critical evaluation stage has shown the suitability of the use of fluorosensing for enhancing clean-in-place monitoring for industrial applications.

## Figures and Tables

**Table 1:** White chocolate composition.

Typical Values	Per 100 g
Fat	33 g
of which saturates	20.2 g
Carbohydrate	61.5 g
of which sugars	61.5 g
Fibre	0.5 g
Protein	4.7 g
Salt	0.2 g

**Table 2:** Nonlinear autoregressive models with exogenous inputs (NARX) Network results.

Training Dataset	Testing Dataset	MSE				
		3HLN	6HLN	10 HLN	15 HLN	20 HLN
D2	D1	9.37 × 10^−3^	4.34 × 10^−4^	3.70 × 10^−3^	2.90 × 10^−2^	5.80 × 10^−3^
D3		1.24 × 10^−5^	7.87 × 10^−7^	**2.72 × 10^−7^**	1.00 × 10^−3^	1.72 × 10^−1^
D1 + D2		5.67 × 10^−3^	1.29 × 10^−3^	1.28 × 10^−1^	1.88 × 10^−2^	6.64 × 10^−2^
D1 + D3		1.11 × 10^−6^	1.36 × 10^−5^	1.40 × 10^−3^	1.55 × 10^−4^	3.40 × 10^−3^
D2 + D3		2.44 × 10^−2^	2.57 × 10^−3^	3.70 × 10^−3^	2.30 × 10^−3^	5.41 × 10^−1^
D1 + D2 + D3		5.66 × 10^−3^	1.81 × 10^−2^	8.33 × 10^−5^	1.56 × 10^−2^	1.73 × 10^−1^
D1	D2	1.74 × 10^−2^	6.07 × 10^−3^	**2.85 × 10^−4^**	4.22 × 10^−2^	3.63 × 10^−1^
D3		4.41 × 10^−2^	5.72 × 10^−2^	1.24 × 10^−1^	8.71 × 10^−1^	8.29 × 10^−1^
D1 + D2		4.19 × 10^−2^	2.38 × 10^−3^	9.02 × 10^−1^	2.48 × 10^−1^	1.02 × 10^−1^
D1 + D3		8.05 × 10^−2^	8.79 × 10^−2^	1.44 × 10^−1^	7.29 × 10^−2^	6.15 × 10^−2^
D2 + D3		4.56 × 10^−2^	4.17 × 10^−2^	1.14 × 10^−2^	3.77 × 10^−2^	4.62
D1 + D2 + D3		7.96 × 10^−2^	6.83 × 10^−2^	1.07 × 10^−2^	5.60 × 10^−1^	3.93
D1	D3	3.44 × 10^−8^	4.38 × 10^−8^	**1.03 × 10^−8^**	1.44 × 10^−7^	4.27 × 10^−5^
D2		3.39 × 10^−3^	1.95 × 10^−4^	4.45 × 10^−4^	1.55 × 10^−5^	3.07 × 10^−5^
D1 + D2		2.12 × 10^−3^	1.57 × 10^−4^	1.46 × 10^−1^	1.05 × 10^−2^	3.31 × 10^−2^
D1 + D3		1.58 × 10^−8^	3.64 × 10^−7^	1.44 × 10^−7^	4.38 × 10^−6^	2.04 × 10^−5^
D2 + D3		3.73 × 10^−3^	5.75 × 10^−4^	3.60 × 10^−3^	1.00 × 10^−3^	2.34 × 10^−1^
D1 + D2 + D3		1.96 × 10^−3^	4.80 × 10^−3^	5.71 × 10^−5^	2.06 × 10^−2^	1.63 × 10^−1^
